# A preliminary investigation of high retinoic acid exposure during fetal development on behavioral competency and litter characteristics in newborn rats

**DOI:** 10.1002/brb3.2253

**Published:** 2021-09-02

**Authors:** Hillary E. Swann‐Thomsen, Valerie Mendez‐Gallardo, Leah R. Kollmeyer, Kira Hunter, Michele R. Brumley

**Affiliations:** ^1^ Department of Psychology Idaho State University Pocatello Idaho USA; ^2^ Department of Psychology Penn State University, State College, Pennsylvania USA; ^3^ Present address: Applied Research Division St. Luke's Health System Boise Idaho USA

**Keywords:** meningomyelocele, neural tube, rats, spinal cord, tretinoin

## Abstract

Myelomeningocele (MMC) is the most common and severe type of spina bifida in which the developing spine and neural tube fail to close during prenatal development. This typically results in a small portion of the lower spinal cord and meninges protruding from the back of the individual, accompanied by severe motor and sensory deficits. In rats, retinoic acid (RA) exposure in high doses during fetal development has been shown to induce morphologic and clinical symptoms similar to humans with MMC. The aim of the current study was to examine litter characteristics and sensorimotor function in MMC‐affected rat pups. Pregnant rats were gavage‐fed 2 ml olive oil or all‐trans RA (40, 45, 50 mg/kg) on gestational day 11. Pups underwent behavioral testing on postnatal day 2. Litter characteristics and newborn sensorimotor function varied across RA doses. Pups prenatally exposed to 45 and 50 mg/kg RA weighed significantly less than olive oil and 40 mg/kg RA pups. Litters exposed to 45 mg/kg RA suffered significantly higher mortality rates compared to other groups. Additionally, bladder function was significantly impaired in pups exposed to 40 mg/kg RA. Sensorimotor function findings demonstrated that for most behavioral assessments there was not a significant difference between control and RA‐exposed subjects. However, pups treated with 40 mg/kg RA showed increased facial wiping, suggesting a hyper‐responsiveness to sensory stimuli. Overall, the findings of the current study provide evidence for a model to examine litter characteristics and behavioral effects as well as morphology.

## 1 INTRODUCTION

Spina bifida is a congenital birth defect in which the developing spine and neural tube do not close properly during prenatal development. Individuals affected by spina bifida experience a range of difficulties dependent upon the location and severity of spinal damage. The spectrum of symptoms ranges from little or no problems to reduced sensation and motor control in the legs and feet, difficulty walking, bladder and bowel dysfunction, sexual problems, obesity, depression, learning disabilities, and latex allergy (Spina Bifida Association, 2015; Woodhouse, 2008). Myelomeningocele (MMC) is the most common and severe form of spina bifida that typically results in a small part of the lower spinal cord and meninges protruding from the back of an individual and severe motor and sensory deficits, including paralysis, below the level of the spinal lesion. Individuals with MMC can exhibit a number of problems in addition to motor and sensory deficits, such as reduced sensation, bladder, bowel and/or sexual dysfunction, obesity, depression, and learning disabilities (Woodhouse, 2008). Thus, while MMC is a neurological disorder that predominantly affects spinal cord function, the effects of MMC can cascade into other impairments impacting quality of life (i.e., obesity, which is very likely related to decreased mobility; Woodhouse, 2008).

Although consumption of folic acid during pregnancy reduces the incidence of MMC, the cause of MMC is currently unknown and there is no way to prevent it. In general, it has been thought that MMC is caused by two primary events, characterized by a “two‐hit hypothesis.” The first hit is the primary, defective spinal cord development, followed by the second hit, damage due to the spinal cord being exposed to amniotic fluid and direct trauma (i.e., hydrodynamic pressure; Adzick, 2010; Danzer et al., 2011 ). Recent findings suggest that in addition to defective development of the spinal cord and resulting damage that the presence of the protruding sac often seen in MMC stretches the nerves resulting in additional damage (Oliver et al., 2020). Newly pioneered techniques in spinal surgery have been used to surgically close the spinal canal in human fetuses (Adzick, 2010) and newborns, thus limiting the development of further infection, such as meningitis, as well as improved functional and motor outcomes, and a reduction in additional surgical procedures (Farmer et al., 2018; Houtrow et al., 2020). However, even after pediatric surgery, individuals still suffer from many of the problems mentioned above and have impaired functional ability. Therefore, finding ways to further improve motor and sensory function in individuals with more severe MMC, especially early in life, remains an important issue. For example, research shows that stepping on a treadmill may facilitate the developmental trajectory of walking in infants with MMC (Lee & Sansom, 2019; Pantall et al., 2012; Teulier et al., 2009). These studies report that infants with MMC respond to treadmill training by demonstrating a locomotor stepping response. Although the evoked response is somewhat different than observed in typically developing infants, an ability to adapt to sensory feedback and training is demonstrated in muscle activation (Pantall et al., 2012), which therefore holds promise for developing activity‐based rehabilitation programs to improve sensorimotor coordination and function.

To further study early presentation of MMC, a prenatal rat model of MMC has been developed (Cai et al., 2007; Danzer et al., 2005). In this model, pregnant rats are gavage fed retinoic acid (RA; 20–70 mg/kg) on embryonic day 10 (E10; birth occurs on E22), at doses between 20 and 70 mg/kg (Cai et al., 2007; Danzer et al., 2005). Around E10, the neural tube begins to close, beginning with the anterior neuropore, thus exposure to RA at or around this timeframe likely disrupts neurulation resulting in neural tube defects (Erb, 2006). RA‐exposed rat fetuses develop morphologic and clinical symptomology very similar to humans with MMC (Cai et al., 2007). For instance, by E22, this included a visible protruding sac or lesion in the lumbar and sacral regions of the spinal cord, disruption of spinal cord tissue, disrupted bladder and bowel function, and a high incidence of clubfoot (Danzer et al., 2007), although some visible defects were visible as early as E14 (Danzer et al., 2011). As with humans with MMC, there appears to be some degree of variability observed in the severity of symptoms. Within litters, some rat fetuses showed more severe spinal damage/symptoms, and others showed less severe damage/symptoms. Danzer et al. (2011) reported varying sensorimotor dysfunction in affected fetuses from E18 to E22, such as increasingly reduced pain responses from E18 to E22. In that study, rat fetuses with MMC showed reduced responses to tail and hindpaw pinching. However, more detailed analyses of motor function were not provided. Using the same rat model of MMC, Wei et al. (2012) showed that fetal exposure to a high dose of RA induces cell death and inhibits cell proliferation in the neuroepithelium of the developing neural tube. Therefore, this prenatal rat model of RA exposure has been used to study both prenatal MMC symptoms and mechanisms of action, and thus has been helpful in understanding more about what may be occurring when spina bifida occurs in humans.

Given the striking similarity between this animal model and the presentation of MMC in humans, there is great potential for this prenatal rat model to be used longer term to investigate postnatal development of sensory and motor function, and recovery of function, following induction of MMC. Previous studies (Cai et al., 2007; Danzer et al., 2005, 2011; Wei et al., 2012) that have used this RA‐exposure MMC model in rats have all examined MMC‐affected rats before or immediately after birth. It was thought that RA‐exposed animals would not be able to survive postnatally (N.S. Adzick, personal communication). However, in order to conduct longitudinal testing, it is important to know if RA‐exposed rat pups will survive after birth (because some of the damage that occurs prenatally can be quite severe). If they survive, what does their behavior look like, and can this model be used to answer questions about long‐term effects of MMC and recovery of function?

Thus, the purpose of the current study was to begin to extend the prenatal rat RA‐induced MMC model into the postnatal period. This preliminary investigation sets out to explore effects of RA exposure in newborn rats. If successful, this model then can be used to explore longer term effects. As part of this, our goal was to document litter characteristics (i.e., mortality rates, body mass, body length) and behavior function for different parts of the body, within 2 days after birth in rats, and to identify a dose of RA for future postnatal studies in rats. Prenatal exposure to RA occurred on embryonic day 11 (E11), with doses piloted out in our laboratory and based on previous studies (see Section 2). After birth, prenatally exposed (to RA or vehicle control) newborn rats underwent a battery of behavioral tests to examine behavioral competency, in addition to litter characteristics that were measured and recorded. It was hypothesized that RA‐treated neonatal rats would show less behavioral competency compared to control rats, as well as differences in litter characteristics, such as body weight, body length, bladder function, and mortality rates. Additionally, we hypothesized that higher doses of prenatal RA exposure would increase behavioral impairments and highlight differences in litter characteristics across doses of RA. Examination of MMC‐affected pups postnatally could help guide future research utilizing this rat model of MMC, as well as inform intervention approaches in humans affected by MMC.

## 2 METHODS

### 2.1 Subjects

A total of 12 Sprague–Dawley rat litters were used in this study. Twelve adult Sprague–Dawley female rats were time mated in the Animal Care Facility at Idaho State University. Adult animals were obtained from Simonsen Laboratories. The day of conception, identified via vaginal smear, was designated embryonic day 0 (E0). On E11, nine dams were gavage‐fed RA (to induce MMC in fetuses); three dams were gavage‐fed olive oil (OO) (control group) (Cai et al., 2007; Danzer et al., 2005). Animals were kept on a 12:12‐h light:dark cycle with food and water available ad libitum. A total of 98 rat pups were used from the 12 litters for this study. Subjects remained in the home cage with the dam, except during testing, which occurred on postnatal day 2 (P2). Animal care and use were in accordance with NIH guidelines and the Idaho State University Animal Care and Use Committee.

### 2.2 RA exposure

RA exposure was used to induce MMC (Danzer et al., 2005, 2007). Pregnant rats were administered a single intragastric infusion of *all‐trans* RA suspended in 2 ml OO. Previous research has shown that a RA dose of 60 mg/kg body weight induces MMC symptoms in greater than 80% of offspring (Danzer et al., 2005); however, preliminary data from our lab found that this dose was lethal, and offspring did not survive until day of testing (P2). Thus, for the current study, dams received a RA dose of 40 mg/kg body weight (RA 40), 45 mg/kg body weight (RA 45), or 50 mg/kg body weight (RA 50). Females in the control group received infusions of OO only. After infusion, the pregnant female was returned to the home cage.

### 2.3 Experimental design

Because some rat pups had a visible lesion on their lower back (which is a characteristic of severe MMC) as well as other slight deformities following RA exposure, it was anticipated that dams might not take care of the pups in their litter. Initially, to account for this possibility and to facilitate postnatal survival, rat pups were delivered via c‐section on E22 (day of birth), and we attempted to artificial rear them using the “pup in the cup” model (Hall, 1975). Interestingly, we found that the pups delivered via c‐section and artificially reared had an increased mortality rate that did not appear to be dependent upon RA treatment (OO = 100% of pups died before testing on postnatal day 2, RA 40 = 100% of pups died before testing, RA 45 = 61% of pups died before testing, RA 50 = 90% of pups died before testing). Due to the high mortality and infection of pups delivered via c‐section and artificially reared, we ultimately decided not to move forward with this methodology and instead focused on litters that were delivered vaginally by the dam and cared for by the dam.

On day of birth (E22/postnatal day 0 (P0)), experimenters recorded the number of rat pups in each litter with minimal disturbing of the home cage and dam. On P2, the number of pups was again recorded (to determine number of pups that died within the first 48 h after birth), and body mass and length (nose to rump distance) of each pup was recorded. Rat pups were examined for any external abnormalities, such as spinal lesions, jaw and mouth defects, and clubfoot, prior to testing. Behavioral testing also occurred on P2. RA‐treated pups were euthanized via CO_2_ and fixed for further external morphology examination, as well internal morphology.

### 2.4 Behavioral testing and scoring

On the day of testing (P2), rat pups were tested inside an infant incubator that controls temperature (35°C) and humidity (70%). Each pup in the litter was tested. Special care was taken to not apply pressure to the back or disturb any lesion site during testing. Cameras located inside the incubator were connected to outside recording units, and all behavioral tests were recorded onto DVD. Behavior was scored using JWatcher software during DVD playback. Behavioral scoring occurred during normal to reduced speed DVD playback for each behavioral test separately. Pups underwent a series of behavioral tests, as described in detail below. Prior to behavioral testing, bladder function was examined. The bladder was expressed by rhythmic stroking stimulation of the anogenital (perineal) region using a small, soft Kimwipe tissue for 1 min. Pups were stimulated up to three times. Mass of the tissue was compared before and after urination to determine the amount of urine released on the first trial where urination was evoked. This method for urination testing was developed and piloted in our lab for the purposes of current study.

#### 2.4.1 Lateral contact righting

Contact righting involves full body (axial) coordination and is used to test vestibular and tactile sensory development (Pellis et al., 1991). Contact righting occurs when the animal positions itself in a prone posture, upon release from an initial starting posture (i.e., side or supine). Righting is an important behavior that rat pups typically show to orient toward the nipple (Eilam et al., 1999). Here, pups were placed onto a flat Plexiglas surface in a lateral (side) position. Each animal was given four trials: two trials that began on the left side and two trials that began on the right side. Each trial was up to 1 min long, with the trial ending at 1 min regardless of if the pup had returned to a prone position or not. There were 30 s between each trial.

Latency to right (establishment of prone posture), strategy, and failure to right were scored for all four 1‐min trials. Latency to right was determined by the delay in seconds from when the pup was released by the experimenter to when the pup established the prone posture. Prone posture (i.e., successful righting) was defined as belly on surface with at least three paws flat on the surface. Righting strategy was divided into three categories: corkscrew, u‐shaped posture, or no strategy (Pellis et al., 1991). Corkscrew righting consisted of the pup rotating their head, neck, and shoulders in one direction while the pelvis rotated in the opposite direction. U‐posture righting consisted of the pups raising their head and limbs upwards from a supine position, forming a “U” shape position before righting. No strategy was coded when the motor pattern used was not corkscrew or U‐shaped posture, and only one strategy was recorded for each pup per trial. Failure to right was recorded as a dichotomous outcome of yes or no for each trial and was determined by a lack of prone posture establishment during the 1‐min trial.

#### 2.4.2 Odor aversion test

Forelimb, as well as hindlimb, responses were used to test limb function. Although it may be counterintuitive to expect differences above the level of the lesion or lower spine, Turner (1985) found differences in hand function in children with MMC compared to children without MMC. Here, forelimb responses were evoked by exposure to lemon extract. A cotton swab was moistened in lemon extract and kept outside the incubator until the first trial. During the trials (two total), the experimenter attempted to make direct contact with the snout and the cotton swab for 15 s. There was a 1‐min observation period after the 15‐s exposure period for both trials.

Behavioral scoring was divided into rostral and caudal passes. The rostral pass captured behavior involving forelimb, head, and trunk movements, which included forelimb responses, such as movement of the forelimbs, paw to snout contact, facial wiping, and forelimb flailing, but also included head turning and pivoting. The caudal pass captured behavior involving hindlimb or whole‐body movements, including backing away from the cotton swab, hindlimb movements, laying on side, rolling, and supination.

#### 2.4.3 Leg extension response

Neonatal rat pups exhibit a bilateral hyperextension of the hindlimbs in response to anogenital licking by the dam, as well as artificial stimulation (Moore & Chadwick‐Dias, 1986; Roberto & Brumley, 2014). This is referred to as a leg extension response (LER). Here, the LER was evoked through rhythmic stimulation of the anogenital region by a soft paintbrush. Pups were held by the experimenter in such a manner as to avoid applying pressure on the back and stimulated for 30 s. Each pup received three trials of stimulation with a 1‐min break between trials. During the break, pups were placed in a prone position on a flat Plexiglas surface. Latency to and duration of bilateral LER were scored for the first trial where the pup exhibited a bilateral LER. The first bilateral LER for each subject was scored.

#### 2.4.4 Tail pinch

Tail pinch was used here to test hindlimb function. Tail pinch in newborn rats reliably evokes a brief bout of alternating hindlimb stepping behavior (Norreel et al., 2003; Swann et al., 2017). In the current study, pups were placed prone on a flat Plexiglas surface, and the tips of miniature forceps were placed laterally on each side of the tail at mid‐length. The forceps were quickly and gently squeezed (Norreel et al., 2003). Pups received a tail pinch, followed by a 1‐min observation period. The 1‐min poststimulus period was scored for hindlimb alternating stepping and nonalternating stepping hindlimb movements, as well as crawling and whole‐body movements.

### 2.5 External and internal morphology

Prior to behavioral testing on P2, RA‐treated subjects were given a visual examination to record external abnormalities and morphology, including protruding spinal lesions, curly tails, shortened tails, limb abnormalities, jaw/oral malformations, and eye and earbud malformations. Preliminary data suggested that there was a high occurrence of the above listed abnormalities that extend beyond spinal lesions (using the 60 mg/kg RA dose); however, lower occurrence was seen in the RA doses used in the present study (40, 45, and 50 mg/kg RA). Thus, external abnormalities were classified as present or absent per subject for analyses.

Immediately after behavioral testing on P2, RA‐treated subjects were euthanized by CO_2_ exposure and preserved in formalin. Gross dissection of the back and spinal cord for RA‐treated subjects occurred under microscopic guidance to indicate the level and extent of any spinal damage. (Due to previous research demonstrating no morphological abnormalities in OO controls, and OO controls were not examined or preserved (Danzer et al., 2005).). Examples of spinal damage recorded included a protruding sac, missing spinal segments, spinal lesions, and discoloration or bruising of the spinal cord. Based on the extent of the damage observed, as well as any external spinal lesions, three groups were categorized. Rat pups that had no visible internal spinal damage or visible external spinal lesion were classified as having no spinal damage; pups that had minimal anatomical damage restricted to 1–2 levels of the spinal cord were classified as having minor spinal damage; and pups that had damage to greater than 2 spinal levels or a protruding sac/external spinal lesion were classified as having major spinal damage.

### 2.6 Data analysis

Data were analyzed using SPSS statistical software (Version 24.0). Data from males and females were pooled together and not examined separately, due to sample size limitations. Analysis of variance (ANOVA) and chi‐square tests were used to examine the effect of prenatal RA exposure on litter characteristics and behavioral competency. When significance was detected, follow‐up tests were conducted and are identified in Section 3. A significance level of *p* < .05 was adopted for all tests.

First, chi‐square tests were conducted for mortality rates from P0 to P2. The number of pups that survived to day of testing was summed for each treatment group. A chi‐square test was performed on the number of alive versus dead subjects for an omnibus comparison of all treatment groups, and then separately for each combination of treatment groups. A series of ANOVAs were conducted to examine the effects of prenatal RA treatment dose on litter characteristics, including pup body mass, length, and bladder function as measured by urine output, as well as external and internal morphology. Prenatal RA treatment dose was the independent variable for each ANOVA. Dependent variables were body mass, body length, urine output, spinal damage and nonspinal morphology. Next, chi‐square tests and a series of one‐way ANOVAs were conducted to examine the effects of prenatal RA treatment on behavioral competency for each behavioral test. Prenatal treatment was the independent variable for each ANOVA. Dependent variables were latency to right (s), righting strategy, and failure to right for the contact righting test. For the odor aversion test, dependent variables included duration of forelimb and hindlimb movement, flailing, facial wiping, head turning, paw to snout contact, and pivoting. Latency to and duration of bilateral LER were the dependent variables for the LER test. Duration of crawling, whole body movement, and hindlimb alternated stepping and nonalternated stepping were the dependent variables included in the analyses for the tail pinch test.

## 3 RESULTS

### 3.1 Litter characteristics

#### 3.1.1 Mortality

A series of chi‐square tests were performed to examine the relationship between prenatal RA treatment and survival rate from P0 to P2. There was a significant relationship between dose of RA and pup survival (*χ* ^2^ (3, *n* ^^= 108) = 21.10, *p* < .001). To further examine which treatment group was driving this significant finding, separate chi‐square tests were conducted for each treatment group combination. Chi‐square tests revealed that there were significant relationships between OO and 45 mg/kg RA (*χ* ^2^ (1, *n* = 53) = 10.48, *p* < .001), 40 mg/kg RA and 45 mg/kg RA (*χ* ^2^ (1, *n* = 62) = 10.95, *p* < .001), and lastly between 45 mg/kg RA and 50 mg/kg RA (*χ* ^2^ (1, 57) = 10.42, *p* < .001). As can be seen in Table 1, rats prenatally exposed to RA 45 showed lower survival rates than OO controls and rats prenatally treated with RA 40 or RA 50. There was not a significant relationship between OO controls and RA 40, OO controls and RA 50, and RA 40 and RA 50, for pup survival.

**TABLE 1:** Mortality rate across retinoic acid exposure conditions

	Survived		Died	
	n	%	N	%
Olive oil	19	90.5	2	9.5
40 mg/kg RA	26	86.7	4	13.3
45 mg/kg RA	15	46.9	17	53.1
50 mg/kg RA	22	88	3	12

Abbreviation: RA, retinoic acid.

#### 3.1.2 Body mass and length

**FIGURE 1:**
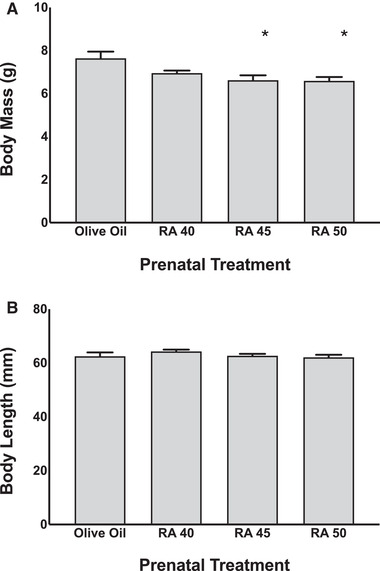
(a) Rat pup body mass at P2, following prenatal exposure to olive oil (control) or dose of retinoic acid (RA 40, 45, or 50 mg/kg). Bars represent means; vertical lines represent SEM. (b) Rat pup body length at P2, following prenatal exposure. Bars represent means; vertical lines represent SEM. Asterisks indicate significant effects

A one‐way ANOVA for pup body mass on P2 revealed a significant main effect of prenatal treatment (*F* (3, 76) = 5.15, *p* < .005), as can be seen in Figure 1A. Tukey's post hoc tests revealed that the P2 body mass for rats exposed to RA 45 (6.62 ± 0.88 g) or RA 50 (6.59 ± 0.79 g, *p* < .01) was significantly lower than OO controls (7.64 ± 1.36 g, *p* < .01). There were no significant differences between OO controls and RA 40 exposed pups, or between any of the RA treatment groups. A one‐way ANOVA for pup body length on P2 did not reveal a significant effect of prenatal treatment (see Figure 1B).

#### 3.1.3 Bladder function

**FIGURE 2:**
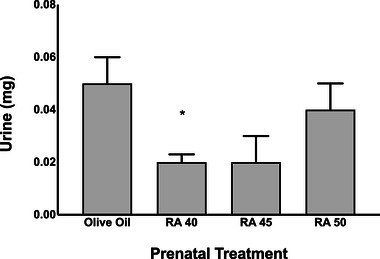
Bladder output (urine) at P2, following prenatal exposure to olive oil (control) or dose of retinoic acid (RA 40, 45, or 50 mg/kg). Bars represent means; vertical lines represent SEM. Asterisk indicates significant effect

**FIGURE 3:**
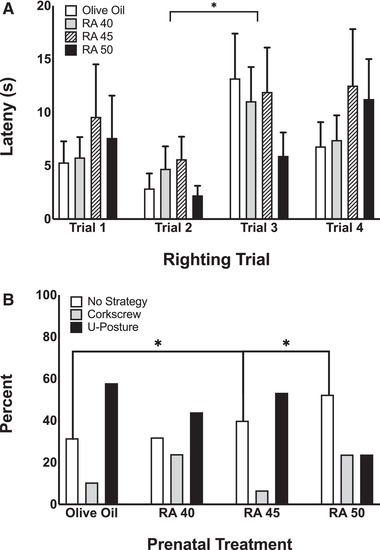
Lateral contact righting measured on P2. (a) Latency to right (i.e., establish prone posture) across trials. Bars represent means; vertical lines represent SEM. (b) Strategy used to right for trial 4 by retinoic acid (RA) treatment. Bars represent frequency. Asterisks indicate significant effects

For bladder function on P2, a one‐way ANOVA revealed a significant main effect of prenatal RA treatment dose (*F* (3,76) = 3.71, *p* < .05). Tukey's post hoc tests revealed that urine output was lower for subjects prenatally exposed to RA 40 compared to OO controls (*p* < .05; Figure 3). There were no significant differences between subjects prenatally exposed to OO versus RA 45 (however, this approached significance at *p* = .058), OO and RA 50, or between RA doses for bladder function as a function of urine output (see Figure 2).

#### 3.1.4 External and internal morphology

A chi‐square test determined that there was not a difference in the presence or absence of external malformations across OO control and RA‐treated subjects, as shown in Table 2. Very few rat pups displayed any external malformations at all (only two pups in the RA 40 group and four pups in the RA 50 group). Similarly, a chi‐square test determined that there was not a difference in the presence or absence of internal morphology across OO control and RA‐treated subjects (see Table 2). Finally, a one‐way ANOVA for the severity of spinal damage was not significant. In the RA 40 group, there were 10 pups with internal malformations (all minor spinal damage), and 10 pups in the RA 45 group with nine minor spinal damage and one pup with major spinal damage, and eight pups in the RA 50 group with minor spinal damage (see Table 2). It is interesting to note that approximately one‐third of all subjects prenatally exposed to RA developed some form of internal spinal damage. Importantly, none of the subjects developed infections, and all subjects completed behavioral testing.

**TABLE 2:** Retinoic acid (RA)‐induced morphology and severity of spinal damage

	External morphology	Internal morphology		
	Present	No spinal damage	Minor spinal damage	Major spinal damage
RA 40 (*n* = 26)	2	16	10	0
RA 45 (*n* = 15)	0	5	9	1
RA 50 (*n* = 20)	4	11	8	0

Abbreviation: RA, retinoic acid.

### 3.2 Behavioral competency

#### 3.2.1 Lateral contact righting

This behavior test was used to assess full body coordination. A repeated measures ANOVA revealed a main effect of time for latency to right (*F* (3, 228) = 4.52, *p* < .01), but not for prenatal RA treatment (see Figure 3A). Paired sample t‐tests indicated that the latency to right from trial 2 (*M* = 3.8 s, SD = 7.7 s) to trial 3 (*M* = 10.4 s, SD = 9.2 s) significantly increased (*t*(41) = 2.55, *p* < .05). A series of chi‐square tests indicated that there were not significant differences in righting strategy across prenatal RA treatment groups for trials 1, 2, and 3. There was a significant difference in righting strategy across prenatal RA treatment groups for trial 4 (***χ*** ^2^ (6, *n* = 79) = 14.23, *p* < .05), such that rats exposed to RA 50 righted using no strategy at a higher frequency than OO controls and rats exposed to RA 45 (Figure 3B). Chi‐square tests for failure to right did not indicate significant differences across treatment conditions.

#### 3.2.2 Odor aversion test

**FIGURE 4:**
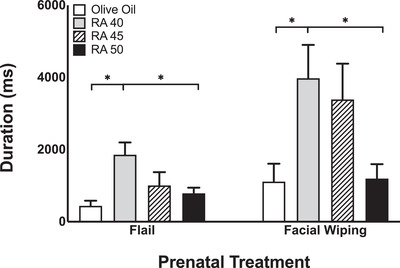
Forelimb responses (flails and facial wiping) to lemon odor exposure. Bars represent means; vertical lines represent SEM. Asterisks indicate significant effects

This behavior test was used to examine motor activity in response to an aversive odor. A one‐way ANOVA revealed a main effect of prenatal RA treatment on forelimb flailing (*F* (3, 77) = 5.44, *p* < .01) and facial wiping (*F* (3, 77) = 4.09, *p* < .01) in response to lemon odor exposure. As can be seen in Figure 4, rats exposed to RA 40 demonstrated longer durations of forelimb flailing and facial wiping compared to OO controls and rats prenatally exposed to RA 50. There were no significant effects of prenatal RA treatment on other forelimb movements, paw to snout contact, head turning, pivoting, backing away from the cotton swab, hindlimb movements, laying on side, rolling, or supination.

#### 3.2.3 Leg extension response

**FIGURE 5:**
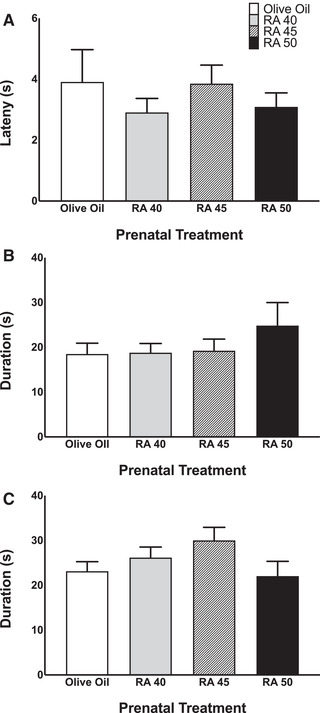
Leg extension response (LER) and tail pinch movement measured on P2. (a) Latency to LER. Bars represent means; vertical lines represent SEM. (b) Duration of LER. Bars represent duration; vertical lines represent SEM. (c) Duration of overall movement following tail pinch stimulation. Bars represent duration; vertical lines represent SEM

This behavior test was used to asses hindlimb coordination. A one‐way ANOVA for latency to bilateral LER was not significant (see Figure 5A); similarly, a one‐way ANOVA for duration of bilateral LER was not significant as shown in Figure 5B.

#### 3.2.4 Tail pinch

This behavior test was used to asses hindlimb function and responsivity of the hind quarters. The duration of overall movement following the tail pinch stimulation was summed across crawling, whole body movements, hindlimb alternated steps, or hindlimb nonalternated steps. A one‐way ANOVA was not significant for overall movement duration following tail pinch (Figure 5C).

## 4 DISCUSSION

The purpose of this study was to examine the feasibility of testing postnatal behavior in rats following prenatal RA exposure. Although the findings reported here represent a preliminary investigation into a sustainable postnatal model of RA‐acid induced MMC, this is the first study to examine behavioral competency of rat pups following prenatal exposure to relatively high doses of RA.

Based on studies using prenatal RA exposure to induce MMC‐like symptoms in rat fetuses, we expected that prenatal RA exposure would induce visible spinal damage and some evidence of external malformations in postnatal rats. Additionally, we were not sure how many rat pups would survive the birth process and even just a couple of days into the postnatal period, given the extent of damage and likely complications that might occur in the nest. Previous studies using RA to induce MMC characteristics reported morphological abnormalities analogous to those seen in humans, such as lesions or protruding sacs on the lower back (Cai et al., 2007; Danzer et al., 2005, 2011; Wei et al., 2012). Although spinal damage did occur in our study as well, there were not significant differences in the presence or absence of external abnormalities or spinal damage across doses of RA, and apparent spinal damage was not the norm. Approximately one‐third of the subjects in the present study had visible spinal damage, and it was not so severe that it prevented behavioral function.

It is possible that the timing of prenatal exposure or RA doses used may have influenced the findings. In a study conducted by Danzer et al. (2005) in which spinal lesions and abnormalities were noted in the majority of fetal rat subjects, a higher dose of RA was used (60 mg/kg) for prenatal exposure. However, this dose was lethal in our pilot work, such that no postnatal rat pups survived until day P2 when prenatally exposed to RA 60 (though many of these subjects did have visible lesions on the back, which is consistent with the Danzer et al.’s study). Thus, we determined that this dose in rats is not a feasible option for developing a postnatal rat model of MMC. Also, Danzer et al. (2005) exposed rat fetuses to RA on E10, whereas we infused dams on E11. In pilot litters of dams infused on E10, we observed high pup mortality rates that seemed to be due to smaller oral cavities in the pups, preventing sufficient feeding as evidenced by the absence of milk bands on the abdomen of pups in the litter. Given the timing of the neural tube closure, E11 still lies within the window for disrupting neurulation; however, it allows the branchial arch and oropharyngeal membrane to form at E10 (Erb, 2006). Thus, the current study opted to infuse a day later than previous studies. Taken together in the context of previous research, the findings from the present study therefore suggest that lower doses of RA are necessary for keeping rat pups alive to the postnatal period, albeit they are likely to result in less severe spina bifida symptoms. Furthermore, given that our morphology assessments were rather gross and unspecific, future work should examine spinal cord morphology (i.e., types of cell damage) to help guide and inform the exact anatomical effects of the RA dose(s) used.

We found that, consistent with our hypothesis, bladder function was impacted following RA exposure. Rat pups exposed to RA 40 had significantly lower urine output compared to OO controls. This observation is consistent with prenatal RA exposure affecting lower spinal cord functions (Cai et al., 2007), as voiding in neonatal rats has been shown to be governed by somato‐bladder spinal reflex pathways (de Groat, 2002). Interestingly, subjects exposed to RA 40 also exhibited significantly higher frequencies of flailing and facial wiping in response to lemon odor exposure but did not show significant behavioral changes in tests of hindlimb activity. This increase in behavioral reactivity is an unexpected direction of effects (i.e., in the forelimbs) and warrants further exploration and replication. Lemon is generally known to be an aversive stimulus to rats and evokes a strong, aversive facial wiping response even in fetal rats 2 days before birth (Brumley & Robinson, 2004). Thus, it is unclear if the findings of increased forelimb reactivity in the current study is an effect having to do with motor coordination or a change in sensory threshold for responding to an aversive stimulus.

Overall, we expected that postnatal litter characteristics and behavioral performance would be impacted by RA dose, with higher doses of prenatal RA exposure having the most significant or deleterious effects. Rats prenatally treated with RA 45 or 50 weighed less than OO controls. Yet, animals treated with RA 50 did not have significantly impacted mortality rates, bladder function, or demonstrate differences in their behavioral repertoire compared to OO control subjects or other RA‐exposed subjects. Although, subjects treated with RA 45 had higher mortality rates compared to controls, as well as other RA‐exposed subjects, they did not exhibit impairments in bladder function (i.e., urine output) or behavioral competency compared to controls. The most prominent behavioral effects were seen in the RA 40 group, as discussed above.

It is curious and interesting that we found no effects related to hindlimb activity. Perhaps this is because rat pups have relatively small hindlimbs at birth, and still are not showing much weight‐bearing movement within the first few postnatal days (Theodossiou et al., 2019). Perhaps if this study were protracted out to a later developmental time point, we would have observed deficits in hindlimb movement, as have been shown in posthatch chicks (Khan et al., 2017).

Overall, findings from the present study suggest that the 40 mg/kg RA dose for prenatal exposure in rats holds the most promise for further developing a small animal postnatal model of RA‐induced spina bifida. Notably, the RA 40 dose had a low mortality rate for newborn rats, allowing for behavioral testing to occur (compared to other RA doses with higher mortality rates or lack of behavioral changes). Although large animal models of spina bifida exist, namely the fetal lamb model (Joyeux et al., 2019), they often require long gestation times and surgical induction of spinal lesions to mimic spina bifida. Recently, a chicken model was developed that exhibited disruptions in leg activity that are consistent with MMC, also providing promise for a new postnatal animal model of spina bifida (Khan et al., 2017). The implications of developing a small postnatal animal model of MMC are enormous, as they would allow for more rapid testing of postnatal outcomes, interventions, and mechanisms of action that could have the potential translate to human outcomes and interventions (i.e., physical therapy approaches). For example, the use of cellular therapies, such as stem cells, will need to be evaluated in animal models before extending such treatments more regularly to humans (Dugas et al., 2020).

Although variable or nonsystematic outcomes may not seem ideal in an animal model, we believe this is actually a strength of the rodent model. The variability of effects captured in this preliminary investigation (i.e., differences in significant findings across different tests for RA groups) is consistent with the variability seen in humans with spina bifida, as well as other animal models (Cai et al., 2007; Danzer et al., 2007, 2011). Although our sample size did not allow us to empirically explore the issue of variability in the current study, anecdotally it was the case that different morphological effects occurred within the same litter. Hence, all individuals with MMC do not present the same. By using a lower dose of RA than in previous studies with rats (Cai et al., 2007; Danzer et al., 2005, 2007, 2011), perhaps this limited lethal and more teratogenic effects in our study; yet, it will permit examination of variability to be explored in future studies. Finally, we must emphasize again that although this postnatal rat model is promising, much work will need to be done in the future to realize the utility and validity of this model, including longer term developmental observations.

## 5 LIMITATIONS

There are additional factors that cannot be ruled out as possible contributors to findings of the current study, including possible changes in maternal behavior and litter differences. In the existing literature, few studies have examined how gestational exposure to RA might impact maternal behavior. Holson et al. (1997) found that cross‐fostered rats treated with low doses of RA had higher mortality rates than cross‐fostered control pups, despite no significant differences in maternal behavior across RA‐treated and control litters. However, there is research to suggest that gestational exposure to retinoids may impact lactation and results in lower rates of pup survival as a result of suppressed lactation (Edelmann et al., 1994). As far as we are aware, previous studies have not specifically examined the maternal behavior of RA‐treated dams and their litters—outside of lactation, and only that of cross‐fostered dams. Closer examination of maternal behavior following gestational RA exposure is warranted to further elucidate these possible associations. Additionally, for the purpose of the current study, each pup within a litter was included in all behavioral tests and measurements. Thus, we cannot rule out the possibility of litter differences as impacting our findings. Future studies should consider the possibility of litter differences as a contributing factor and control for potential litter effects.

## 6 CONCLUSION

Continued research on spina bifida, and particularly MMC, have focused on prenatal repair of lesions and potential impacted mechanisms and physiological processes that result in affected behavior. As of yet, few animal studies have examined postnatal behavioral competency to further advance our understanding of how early behavioral interventions may recover behavioral and sensory function in individuals with spina bifida. Findings from the current study recommend the use of 40 mg/kg of RA in the prenatal exposure model for inducing MMC in postnatal rats, as it results in changes in bladder output and behavioral reactivity but does not significantly impact survival rate or pup growth in the immediate postnatal period. This study provides important preliminary evidence to support the viability of future longitudinal studies examining behavioral changes in postnatal rats following a prenatal insult due to RA exposure. We posit that exposure to RA during the embryonic periods creates a behavioral model of postnatal function that reflects the epidemiological range of effects of spina bifida seen in humans.

### PEER REVIEW

The peer review history for this article is available at https://publons.com/publon/10.1002/brb3.2253

## Data Availability

The data that support the findings of this study are available from the corresponding author upon reasonable request.
